# Pre-existing T cell immunity determines the frequency and magnitude of cellular immune response to two doses of mRNA vaccine against SARS-CoV-2

**DOI:** 10.1016/j.jvacx.2022.100165

**Published:** 2022-05-02

**Authors:** José L. Casado, Pilar Vizcarra, Johannes Haemmerle, Héctor Velasco, Adrián Martín-Hondarza, Mario J. Rodríguez-Domínguez, Tamara Velasco, Sara Martín, Beatriz Romero-Hernández, Marina Fernández-Escribano, Alejandro Vallejo

**Affiliations:** a Department of Infectious Diseases, Hospital Universitario Ramon y Cajal, Madrid, Spain; b Department of Prevention of Occupational Risks, Hospital Universitario Ramon y Cajal, Madrid, Spain; c Laboratory of Immunovirology, Department of Infectious Diseases, Health Research Institute Ramon y Cajal (IRyCIS), Hospital Universitario Ramon y Cajal, Madrid, Spain; d Department of Microbiology, Health Research Institute Ramon y Cajal (IRyCIS), CIBERESP, Hospital Universitario Ramon y Cajal, Madrid, Spain

**Keywords:** SARS-CoV-2 humoral response, Cellular response, mRNA vaccine, Pre-existing immunity, COVID-19, Coronavirus disease 2019, S protein, Spike protein, SARS-CoV-2, Severe acute respiratory syndrome coronavirus 2, RT-PCR, retrotranscriptasepolymerase chain reaction, CLIA, chemoluminiscent immunoassay, IgG, immunoglobulin G, AU, arbitrary units

## Abstract

Little is known about the factors associated with lack of T-cell response to mRNA vaccines against SARS-CoV-2. In a prospective cohort of 61 health care workers (HCWs), 21% and 16% after the first dose of mRNA BNT162b vaccine, and 12% and 7% after the second dose, showed lack of CD4^+^ and CD8^+^ T-cell response, respectively. Pre-existing T-cell immunity, due to past infection (46%) or cross-reactive cellular response (26%), was significantly associated with T-cell response in frequency (CD4+ T-cell, 100% vs 82% after two doses; p = 0.049) and in the magnitude of T-cell response during follow up. Furthermore, baseline CD4^+^ T-cell correlated positively with the titer of specific IgG-antibodies after first and second vaccine dose. Our data demonstrate that cross-reactive T-cells correlate with a better cellular response as well as an enhanced humoral response, and we confirm the close correlation of humoral and cellular response after mRNA vaccination.

## Highlights

• Lack of CD4 T-cell response to first/second dose of mRNA vaccine was observed in 21% and 12% of health care workers (HCWs).
• T-cell response was greater in frequency/magnitude in HCWs with pre-existing immunity, due due to infection or cross-reactivity.
• Pre-existing immunity correlated with the magnitude of specific antibodies production after vaccination.

## Introduction

Current mRNA BNT162b2 vaccine reported high efficacy in preventing symptomatic SARS-CoV-2 infections after two doses[1], [2], but the details of T-cell response following vaccination are still incompletely understood, and questions remain about the correlation between humoral and cellular response[3].

Indeed, T-cells are involved in the early identification and clearance of viral infections and also support the development of antibodies by B cells[4]. Moreover, T-cell responses are not significantly disrupted by the variants of concern[5] and they can contribute reducing COVID-19 severity[6]. Thus, measurement and quantification of T-cell responses will be key to identify factors associated with lack of response, to establish correlates of protection, and to understand the need of additional vaccine doses. Furthermore, pre-existing cellular response by past infection or cross-reactivity with other coronaviruses might be of importance to achieve a greater and durable immune response after vaccination[7], an important issue since pre-existing T-cell response to SARS-CoV-2 has been observed in 30–60% of unexposed individuals [8], [9].

To clarify the differences in cellular response to the two doses of vaccine, and to identify the factors associated with a lower response both in rate and magnitude, we analyze sequentially the T-cell immune response in previously infected and uninfected health care workers (HCWs) after two doses of the Pfizer/BNT162b2 mRNA vaccine.

## Material and methods

Sixty-one HCWs evaluated 3 months before vaccination (median 147 days, IQR, 133–160) in a cross-sectional study about humoral and T-cell response to SARS-CoV-2 underwent blood analysis at least 17 days after the first and after the second dose of BNT162b2 vaccine. The participants were divided in convalescents (26, 43%) with clinical or/and serological evidence of previous SARS-CoV-2 infection, and infection-naïve HCWs (35, 57%), who had confirmed negative serology at inclusion, and did not refer previous suggestive symptoms (fever, cough, anosmia, ageusia, headache, diarrhea) or a positive RT-PCR/serology. Both at inclusion and before vaccination, participants were tested for anti-SARS-CoV-2 IgG antibodies to SARS-CoV-2N protein (COVID-19-SARS-CoV-2 IgG ELISA, Demeditech, Germany) to confirm serologic status and rule out subclinical infections, as this was the test used for diagnosis. After each dose of vaccination, humoral response to the S domain of the spike protein was quantified through SARS-CoV-2 IgG II Quant Alinity **(**Abbott, Maidenhead, UK; positivity threshold 50 arbitrary units (AU)/ml; upper limit 40,000 AU/ml).

### T-cell immune response

Briefly, overlapping peptides spanning the immunogenic domains of the SARS-CoV-2 spike (S) protein were used to stimulate peripheral blood mononuclear cells (PBMCs) from the participants (PepTivator SARS-CoV-2 Prot S, Miltenyi, Germany) followed by the quantitation of specific interferon (IFN)-γ-producing CD4^±^ and CD8^±^ T-cells (Rapid Cytokine Inspector CD4^±^/CD8^±^ T cell kit, Miltenyi, Germany) by multiparametric flow cytometry on a MACSQuant Analyzer 10 using MACSQuantify software. A detailed description of the Methods used is included as supplementary file (Supplementary Methods).

This study was approved by our IRB (EC162/20) and written informed consent was obtained from all participants.

### Statistical analysis

Cellular response was analyzed globally and according to the presence of prior T-cell immunity. Comparisons between groups were performed using two-tailed statistical tests, chi-square or Fisher’s exact tests for categorical variables, and Mann-Whitney test or 1-way analysis of variance (Kruskal-Wallis test) with Dunn’s correction for multiple comparisons, as appropriate. Correlation between quantitative variables was studied using Spearman rank-order correlation test. Statistical significance was defined as two-sided p values < 0.05. Statistics were performed with SPSS, v 23.0.

## Results

Characteristics of enrolled individuals are shown in Table1. As mentioned, we distinguished two groups: 26 convalescent HCWs (42%) and 35 infection naïve HCWs. Just before vaccination, IgG against protein N continued to be positive in 12 out of 26 (46%) convalescent HCWs. Thus, to highlight, all the included HCWs had 2–3 consecutive serological determinations for a correct categorization (for diagnosis only in those with past infection, and at the time of inclusion and pre-vaccination in all the cases).

**Table 1:** Baseline characteristics of the 61 health care workers included.

	**Convalescent HCWs** N = 26	**Uninfected HCWs** N = 35
Age (years)	54 (41–61)	52 (41–57)
Sex (Female n, %)	16 (62%)	24 (69%)
Body Mass Index (Kg/m^2^)	24 (22.4–27)	23.4 (21.3–25.4]
Comorbidities (n, %) Hypertension Diabetes	3 (11%) 1 (4%)	4 (11%) 2 (6%)
Working at COVID ward Time working at COVID ward, weeks History of (n, %): Positive SARS-CoV-2 RT-PCR	15 (58%) 6 (4–9) 21 (81%)	20 (57%) 10 (4–12) -
Positive anti-N SARS-CoV-2 IgG	16 (61%)	–
Time from		
Inclusion to vaccination (days)	153 (138–161)	145 (127–155)
Infection to vaccination (days)	327 (319–335)	
Pre-vaccine:		
Positive IgG N-specific antibodies Median value (RU/mL)	12 (46%) 10,1 (4.8–24.2)	0 4,75 (3,7–6,2)
CD8 + T cell response to S (n, %) CD4 + T cell response to S (n, %)	12 (46%) 11 (42%)	9 (26%) 7 (20%)

Values are expressed as median, interquartile range unless otherwise explained; HCW, health care workers; RT-PCR, real time polymerase chain reaction; RU, relative units.

Pre-existing T-cell immunity against protein S was observed in 21 individuals (34%): 8 out of the 12 HCWs with positive serology (67%), 4 out of the 14 convalescents with negative serology at baseline (28%), and 9 out of the 35 infection-naïve (26%), the latter cases attributed to cognate cross-reactivity. Nevertheless, the magnitude of CD4^+^ T-cell response was greater in convalescent with positive serology in comparison with those with past infection who had negativized serology before vaccination (p = 0.013). Of note, a similar magnitude of T-cell response after stimulation was observed between convalescent and infection-naïve individuals with cross-reactivity (**Supplementary** Fig. 1**).** These differences in baseline humoral and T-cell immunity permit us to separate our cohort in 4 different subgroups: convalescent with/without positive serology, and infection-naïve individuals with/without cross-reactivity.

### IgG Spike-specific humoral response

**Fig. 1:**
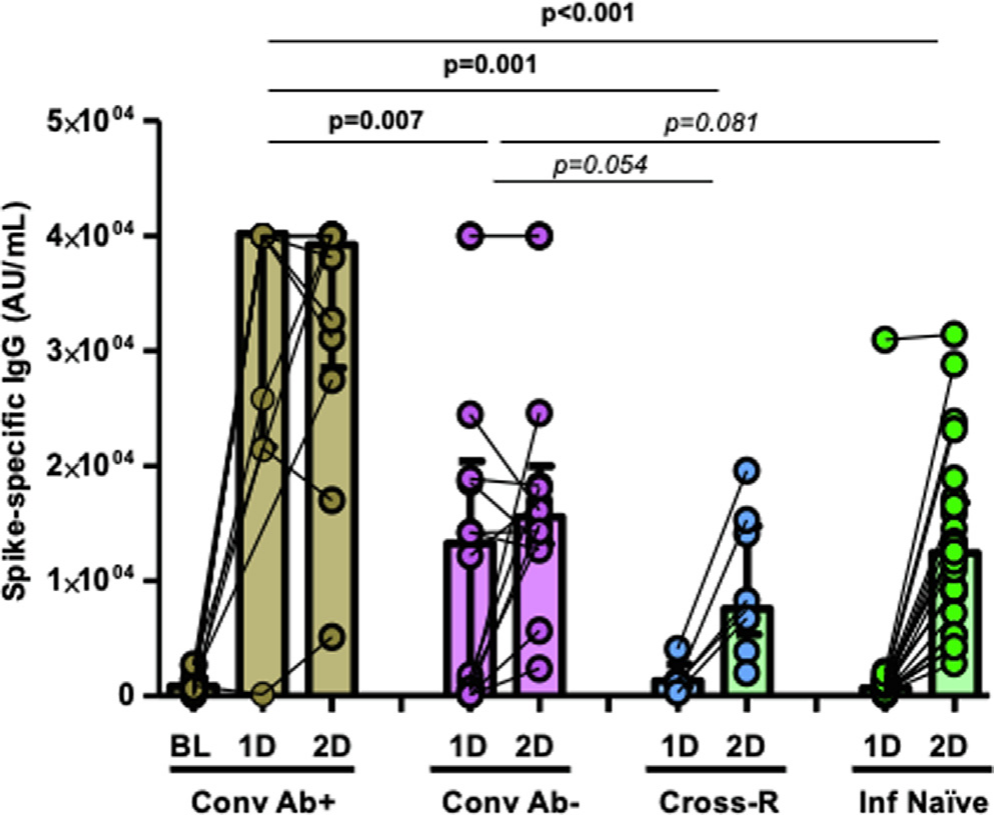
S-specific IgG antibody responses after first (1D) and second dose (2D) of BTN162b2 mRNA vaccine against SARS-CoV-2 (limit of detection, 50 AU/mL). Comparisons between convalescent HCWs with persistence of antibodies (Conv Ab+, brown bars, including baseline S-specific IgG antibodies pre-vaccination, BL), recovered HCWs without antibodies pre-vaccination (Conv Ab−, purple bars), infection-naïve individuals with cross-reactive T-cells (Cross-R, blue bars), and infection-naïve individuals without cross-reactive immunity (Inf Naïve, green bars). Each dot represents an individual after the first and second dose of vaccine. Boxes represent median and interquartile range. Lines between points indicate individual changes for the HCWs involved.

By considering a limit of 50 AU/mL, spike IgG-specific humoral response was observed in all the cases after the first dose (geometric mean concentration, GMC; 1,481.1 AU/mL; interquartile range, IQR, 486.8–23037.5), and overall, it was increased by 10-fold after the second dose (GMC, 14,326.3 AU/mL; IQR 8,656.5–24,231.6), an increase observed almost exclusively in uninfected participants**.** There were no differences in humoral response according to age, presence of comorbidities, or body mass index (BMI). Nevertheless, convalescent HCWs with pre-existing N-specific IgG response showed the highest levels of S-specific IgG antibodies since the first dose of vaccine (GMC; 40,000 and 39,077 AU/mL in both determinations) compared to convalescent HCWs who lost the antibodies against the virus (13,183 and 15,497 AU/mL; p < 0.01 in both determinations in comparison with the convalescent with antibodies pre-vaccination), whereas infection-naïve individuals reach a 12-fold significant increase only after the second dose (median 994 and 11,726 AU/mL; p < 0.01 in both comparisons compared to convalescent individuals with positive serology; Fig. 1**)**.

### T-cell response

Of note, CD4^+^ or CD8^+^ T-cell response after the first dose of vaccine were highly correlated (rho = 0.881; p < 0.01). At this moment, lack of CD4^+^ or CD8^+^ T-cell response was observed in 13 (21%) and 10 (16%) individuals, respectively. Pre-existing immunity was the main factor associated with T-cell response and only 1 case of no response was observed among the 21 individuals with pre-existing immunity secondary to past infection or cross-reactivity (1/21, 5% vs 12/40, 30%; p = 0.082). Thus, one third of infection-naïve HCWs without cross-reactivity had a lack of CD4^+^ T-cell response to the first dose of vaccine. Also, a similar lack of CD8 + T-cell response was observed in those with pre-existing immunity vs infection-naïve individuals, albeit it was not significant (1/21, 5% vs 9/40, 23%; p = 0.123).

**Fig. 2:**
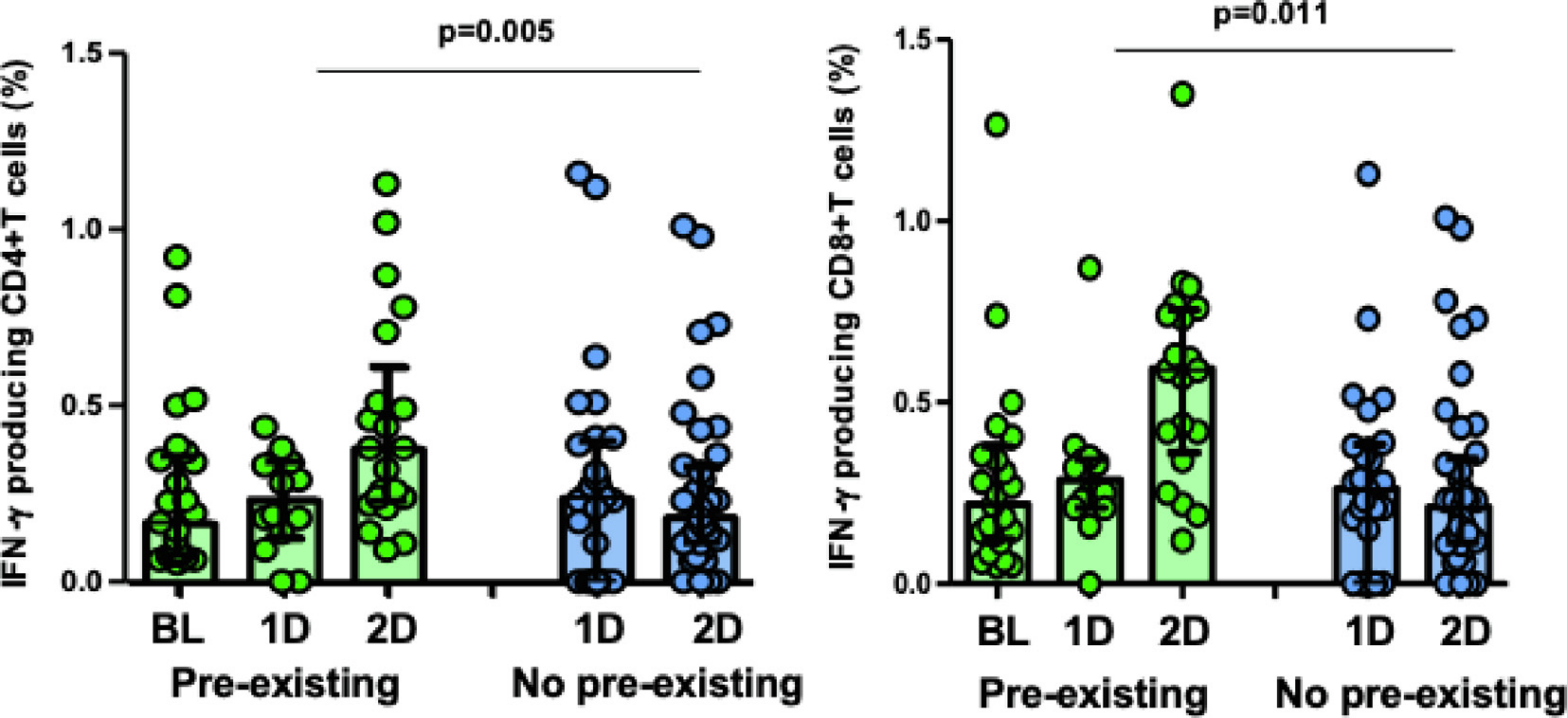
IFN-γ producing CD4+ and CD8+T cells against S peptides pre-vaccination (BL), and after the first (1D) and second dose (2D) of BNT162b2 vaccine, according to pre-existing immunity (green bars) or not (blue bars) in healthcare workers (HCWs). Each dot represents an individual prevaccination or after the first and second dose of vaccine. Boxes represent median and interquartile range.

Only 7 (12%) and 2 (3%) individuals remained with lack of S-specific CD4^+^ or CD8^+^ T-cell response after the second dose of the vaccine, respectively. However, as expected, these individuals were infection-naïve without cross-reactivity (18% and 5%, respectively) whereas all the patients with pre-existing immunity had CD4^+^ and CD8 + T-cell response to the second dose of the vaccine (p = 0.042). Furthermore, pre-existing CD8^+^ and CD4^+^ T cell responses to S protein were further augmented by vaccination and it determines the magnitude of T-cell response after the second dose (CD8^+^, p = 0.011; CD4^+^, p = 0.005; Fig. 2**).** Notably, those without T-cell response to the first dose of vaccine had a lower cellular response to the second dose, which reach statistical significance for CD8^+^ T cells (Fig. 3A**).**

**Fig. 3A:**
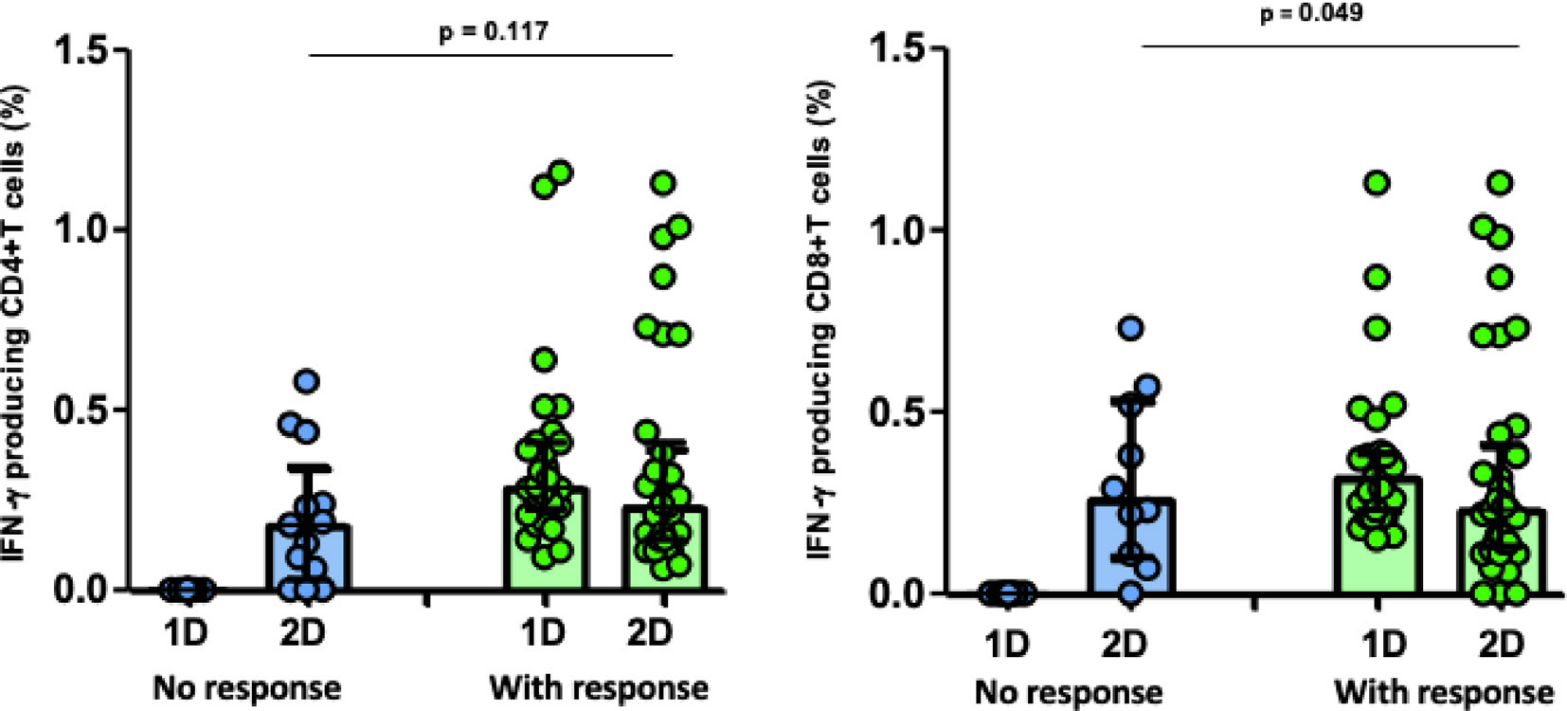
IFN-γ producing CD4+ and CD8+T cells against S peptides after the first (1D) and second dose (2D) of BNT162b2 vaccine in healthcare workers (HCWs), according to lack of cellular response (blue bars) or T-cell response (green bars) to the 1st dose of vaccination.

We evaluated other clinical factors that could contribute to lack of T-cell response among those without pre-existing immunity. Thus, individuals without CD8^+^ T-cell response after the first dose were predominantly older (53 vs 47 years; p = 0.11) and male (p = 0.065) albeit it was not statistically significant.

### Correlation between humoral and cellular response

Before vaccination, pre-existing CD8^±^ and CD4^±^ were weakly but significantly correlated with specific antibodies pre-vaccination (CD8^±^, rho = 0.278, p = 0.03) but this correlation with the humoral response was stronger after the first dose (CD4^±^, rho = 0.486, p < 0.001). Furthermore, humoral response after the second dose continue to correlate with CD4^±^ T-cells at baseline (rho = 0.413; p = 0.004). As expected with this significant correlation, those individuals without pre-existing immunity and who did not develop T-cell response after the first dose of the vaccine had a lower titer of antibodies after the first dose (5,314 vs 14,159 AU/mL; p = 0.021) and after the second dose (10,054 vs 20,322 AU/ml; p = 0.024; Fig. 3B**).**

**Fig. 3B:**
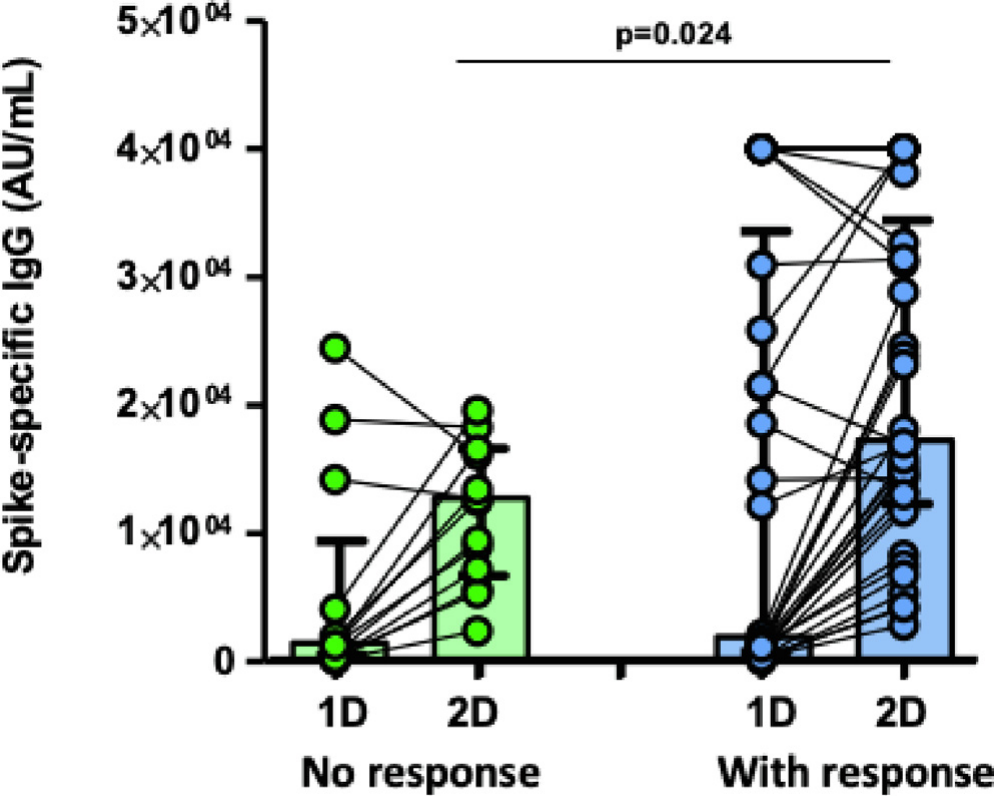
S-specific antibody responses after first (1D) and second dose (2D) of BTN162b2 mRNA vaccine against SARS-CoV-2 (limit of detection of 50 AU/mL). According to lack of T-cell response (green bars) or no response (blue bars) to the first dose. Each dot represents an individual after the first and second dose of vaccine. Boxes represent median and interquartile range. Lines between points indicate individual changes for the HCWs involved.

Thus, in summary, infection-naïve individuals without pre-existing cross-reactive immunity had a significantly lower humoral response to first and second dose (p < 0.01), and they had a lower rate of CD4 + and CD8 + T-cell response to two vaccine doses both in magnitude and in rate of response (30% and 23% after the first dose, 18% and 5% after second dose, respectively), in comparison with the stronger response observed in all the individuals with pre-existing immunity.

## Discussion

We evaluated the CD4^+^ and CD8^+^ T-cell responses induced by SARS-CoV-2 mRNA vaccination in a well-studied cohort of SARS-CoV-2 naïve and recovered HCWs. Our data demonstrated the importance of pre-existing immunity, secondary to past infection or due to cross-reactivity, to determine the frequency and magnitude of T-cell response after one or two doses of vaccine[10].

In our study, 30% and 18% of individuals without pre-existing immunity showed lack of CD4^+^ T cell and lower magnitude of response to the first and second dose of the vaccine, a fact that could contribute to a weaker or shorter immune response[11]. This could be important, as antigen-specific memory CD4^+^ and CD8^+^ T cells are likely to be less impacted by antibody escape mutations in variant viral strains[5], [12]. A similar overall rate of no cellular response has been observed in other studies. Sahin described a 94% of T-cell response after two doses of mRNA vaccine[13]. Furthermore, in a similar study, Painter observed 67% and 85% of CD8 + T-cell response after the first and second dose of vaccine in infection-naïve individuals without T-cell response at baseline. [14].

Moreover, we demonstrated that pre-existing cross-reactive T-cells correlated positively with the induction of S-IgG antibody titers after the first and second dose of the vaccine. These data are not surprising, since antigen-specific CD4^+^ T-cell response plays an important role in antigen-specific B cell development, maturation and survival [14], [15], highlighting the convergent development of the humoral and cellular adaptive immunity [16].

Thus, cross-immunity may be responsible for the unexpectedly high efficacy of current vaccines even after a single dose [7], as it has been demonstrated a role in decreasing the severity of infection [6], [17]. Also, pre-existing T-cell immunity could clarify some controversial data about the correlation between humoral and cellular response, especially in cross-sectional studies, due to the wide heterogeneity in the magnitude of individual spike-specific T cell responses [3]. Furthermore, HCWs without CD4^+^ T-cell response after the first dose and subsequent blunted cellular and humoral response after the second dose could be the best candidates for additional vaccine doses.

We tried to identify other factors associated with lack of T-cell response. Vaccine-induced B cell and antibody responses have been noted to decrease with age[18]. Also, magnitude and quality of SARS-CoV-2 cross-reactivity declined with age, suggesting a possible role for age in decreasing response to the vaccine[10]. However, substantial age-associated changes in the induction of antigen-specific T cell responses have not been previously observed[14]. We observed a trend for a worse response associated with older age and sex male, but we consider that we do not have enough sample size to be able to correctly assess this question.

Limitations of our study include the small sample size. Also, time from initial T-cell evaluation (inclusion) to vaccination was around 3 months, and we cannot preclude changes in T-cell cross-reactive immunity during this period. In addition, asymptomatic infections and misclassification of cross-reactivity in the infection-naïve group was possible but unlikely because of the high sensitivity of repeated serological test at least in two determinations, at inclusion and pre-vaccination, and the similar humoral response observed in infection-naïve individuals with and without cross-reactivity. Finally, we did not have important data about incident infections in this cohort, and therefore we cannot establish the risk of disease associated with no response or with a weaker T-cell response.

In conclusion, we demonstrate that pre-existing T-cells correlate with a better cellular response as well as an enhanced humoral response. Both T-cell response and humoral response were correlated following mRNA vaccination, and those infection-naïve HCWs without cellular immune response to the first dose had a weak cellular and humoral response after two doses. It remains to be determined the specific T cell response that can protect individuals against COVID-19. Also, further studies should determine the duration of clinical protection in both convalescent and infection-naïve individuals.

## CRediT authorship contribution statement

**José L.Casado:** Conceptualization, Data curation, Formal analysis, Funding acquisition, Investigation, Methodology, Project administration, Resources, Software, Supervision, Validation, Visualization, Writing – original draft, Writing – review & editing. **Alejandro Vallejo:** Conceptualization, Data curation, Formal analysis, Funding acquisition, Investigation, Methodology, Project administration, Resources, Software, Supervision, Validation, Visualization, Writing – original draft, Writing – review & editing. **Pilar Vizcarra:** Data curation, Investigation, Methodology, Validation. Johannes Haemmerle: Data curation, Investigation, Methodology, Validation. **Héctor Velasco:** Data curation, Investigation, Methodology, Validation. **Adrián Martín-Hondarz:** Data curation, Investigation, Methodology, Validation. **Mario J. Rodríguez-Domínguez:** Data curation, Investigation, Methodology, Validation. **Tamara Velasco:** Data curation, Investigation, Methodology, Validation. **Sara Martín:** Data curation, Investigation, Methodology, Validation. **Beatriz Romero-Hernández:** Data curation, Investigation, Methodology, Validation. **Marina Fernández-Escribano:** Investigation, Methodology, Supervision, Writing – review & editing.

## Declaration of Competing Interest

The authors declare that they have no known competing financial interests or personal relationships that could have appeared to influence the work reported in this paper.
