# Supplementary material for: Pre-existing T cell immunity determines the frequency and magnitude of cellular immune response to two doses of mRNA vaccine against SARS-CoV-2
Source: Vaccine X. 2022 May 2;11:100165. doi: 10.1016/j.jvacx.2022.100165 (PMC9057925; doi:10.1016/j.jvacx.2022.100165)
Supplement: Supplementary data 1 [file mmc1.pdf]

Supplementary Methods

The presence of cellular immune response was assessed at the same time points that humoral determinations. SARS-CoV-2-specific CD4+ and CD8+ T-cells were measured using *in vitro* stimulation with SARS-CoV-2 peptide pools of viral proteins encompassing the spike (S), followed by quantitation of CD4+ and CD8+ T-cell specific interferon (IFN)-γ in live cell flow cytometry, using peripheral blood mononuclear cell (PBMC) samples from all subjects. It was considered significantly reactive if the proportion of positive cells in stimulated wells was at least 2-fold higher in comparison with the negative control wells (unstimulated).

In detail, EDTA-blood samples were collected from all individuals. After centrifugation at 200g for 10 min, plasma fraction was collected and again centrifuged at 1200g for 15 min, aliquoted and stored at -80°C. The cellular fraction was diluted with phosphate-buffered saline (PBS) and subjected to Ficoll density gradient centrifugation at 500g for 20 min. PBMCs were washed and frozen in fetal bovine serum (FBS) with 8% dimethyl sulfoxide (DMSO, Sigma, USA) in liquid nitrogen. PBMCs were thawed and plated in 96-well flat-bottom plates at 10^6 cells/well in RPMI-1640 culture medium (Gibco, USA) supplemented with 10% human serum (AB serum, Sigma), 100 IU of penicillin/streptomycin/mL (Gibco, USA), 2 mM L-glutamine, and after 24 hours cells were stimulated in five different conditions in the presence of 1 μg/ml purified anti-CD28 antibody (Miltenyi, Germany). One well was stimulated with the SARS-CoV-2 peptide pools S at a concentration of 1 μg/ml, composed of 15-mer sequences with 11 amino acids overlap, covering the immunodominant sequence domains of the surface glycoprotein S (PepTivator SARS-CoV-2 Prot S, Miltenyi-Biotec, Cologne,

Germany). In addition, one well was cultured with complete medium as an unstimulated, and other well was stimulated adding 1.5 mg SEB (staphylococcal enterotoxin B, Sigma, Germany) as the positive control. An unresponsive sample to SEB would be excluded from the analysis.

Stimulated PBMCs were incubated for two hours before adding brefeldin A (Rapid Cytokine Inspector CD4/CD8 T cell kit, Miltenyi, Germany) into the medium to stop cytokine release and kept in culture for other 14 hours. After incubation, staining of the cells was carried out with the following fluorochromes-conjugated antibodies using Rapid Cytokine Inspector CD4/CD8 T cell kit (Miltenyi, Cologne, Germany): CD3-VioBlue, CD4-APC, CD8-FITC, CD14-PerCP, CD20-PerCP, IFN-γ-PE, and FcR blocking reagent. To exclude dead cells, viability 405/520 fixable dye staining (Milteny, Cologne, Germany) was added for the last 10 min of incubation. Fixation and permeabilization were performed according to the manufacturer´s protocol. Samples were measured and analyzed by flow cytometry on a MACSQuant Analyzer 10 using MACSQuantify software. Cells were analyzed and gated with the following strategy. Single (FSC-A/FSC-H dot plot) and live cells were first selected. Cell debris, monocytes, and B cells were excluded from the analysis with CD14- and CD20-PerCP antibodies. Then, lymphocytes were selected with a FSC-A/SSC-A dot plot, and CD3 T cells were gated. IFN-γ expression was finally analyzed for CD4+ and CD8+ T-cells, and expressed by percentage. It was considered significantly reactive if the proportion of positive cells expressing IFN- γ in stimulated wells was at least 2-fold higher in comparison with the negative control wells (unstimulated), as mentioned.
