# Supplementary material for: Pre-existing T cell immunity determines the frequency and magnitude of cellular immune response to two doses of mRNA vaccine against SARS-CoV-2
Source: Vaccine X. 2022 May 2;11:100165. doi: 10.1016/j.jvacx.2022.100165 (PMC9057925; doi:10.1016/j.jvacx.2022.100165)
Supplement: Supplementary data 2 [file mmc2.pdf]

Supplementary Figure 1

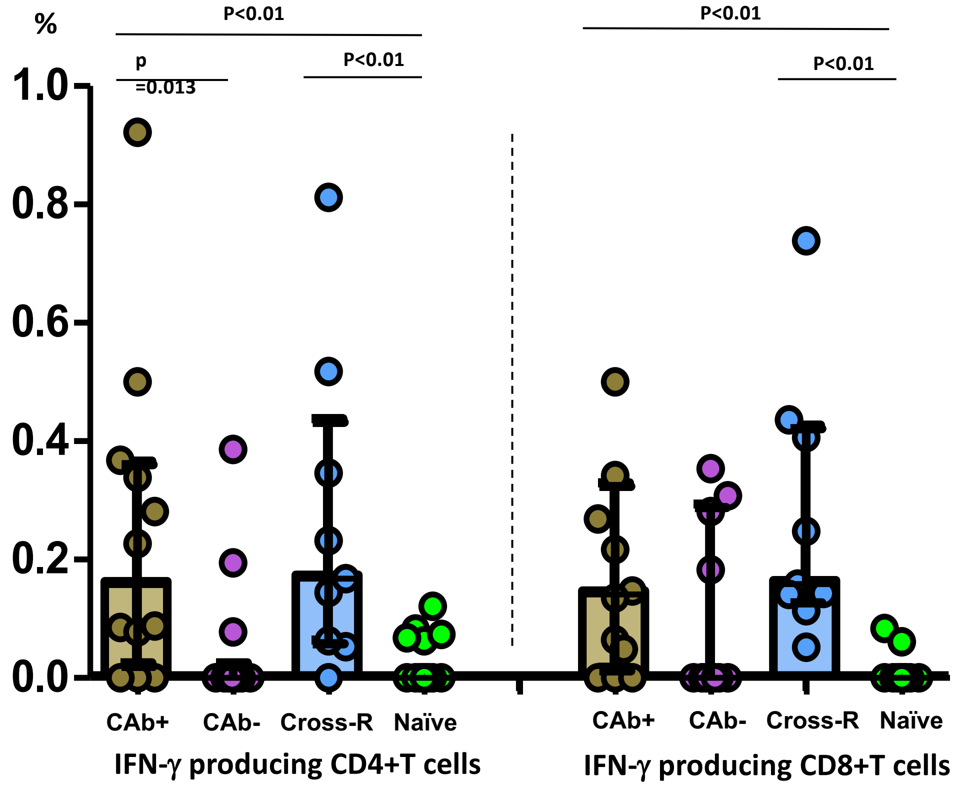

Supplementary Figure 1. Magnitude of CD4+ and CD8+ T-cell response toward viral protein in convalescent HCWs with positive serology pre-vaccination (Cab+, brown bars), convalescent with negative specific serology (Cab-, purple bars), infection-naïve HCWs with cross-reactive immunity (blue bars), and infection-naïve not showing cross-reactivity (green bars).
